# Treatment of bilateral idiopathic trigeminal neuralgia by radiofrequency thermocoagulation at different temperatures

**DOI:** 10.1097/MD.0000000000004274

**Published:** 2016-07-22

**Authors:** Peng Yao, Tao Hong, Zhi-bin Wang, Jia-ming Ma, Yong-qiang Zhu, Hong-xi Li, Yuan-yuan Ding, Chang-lin Jiang, Shi-nong Pan

**Affiliations:** a Department of Pain Management, Shengjing Hospital of China Medical University, Shenyang.; b Department of Pain Management, Daqing Oilfield General Hospital, Daqing; c Department of Radiology, Shengjing Hospital of China Medical University, Shenyang, China.

**Keywords:** idiopathic trigeminal neuralgia, radiofrequency thermocoagulation, recurrence rate, complications, satisfaction

## Abstract

Radiofrequency thermocoagulation (RFT) is an effective treatment for trigeminal neuralgia, but consensus regarding an optimal treatment temperature is lacking. While treatment temperatures ranging from 60°C to 95°C have been reported, RFT at too high a temperature is often followed by serious complications, and comparative evaluations of RFT at different temperatures in a single study are rare.

This current prospective cohort study was to compare immediate and long-term outcomes of RFT at varying temperatures in patients with bilateral idiopathic trigeminal neuralgia (ITN) of maxillary division of trigeminal nerve (V2), mandibular division of trigeminal nerve (V3), and V2+V3, including pain relief, complications, recurrence rate, and patient satisfaction. From May 2011 to April 2016, 62 consecutive patients with bilateral ITN of V2, V3, and V2+V3 were enrolled in the study. These patients underwent bilateral RFT at 68°C and 75°C, respectively, using the same RF parameters. Side-to-side results, including pain relief, complications, and patient satisfaction, were compared during a 5-year follow-up period.

Overall pain relief was satisfactory after RFT. The rate of pain relief after treatment at 75°C was slightly higher than at 68°C (*P* > 0.05). The pain-free rate was 95.1% at 75°C and 93.5% at 68°C at 1 year, 84.3% and 78.1% at 3 years, and 80.7% and 74.4% at 5 years. There were 10 and 13 cases of recurrence, respectively, and 6 cases of bilateral recurrence. The incidence and severity of complications were greater at 75°C (*P* < 0.05) than at 68°C, and therefore the patient satisfaction at the higher temperature was lower (*P* < 0.05).

Patients with bilateral ITN who underwent RFT at different temperatures had consistent pain relief after RFT at both 75°C and 68°C, but there were fewer and less severe complications at 68°C, which was accompanied by greater patient satisfaction. This suggests that RFT at lower temperatures may be preferable, and that a temperature of 68°C can be recommended.

## 1 Introduction

Trigeminal neuralgia (TN) is a clinically common painful disease with an annual incidence of 5–28.6/100,000.^[1–3]^ Patients with TN often experience intense pain that can profoundly diminish their quality of life. Radiofrequency thermocoagulation (RFT) is an efficient, minimally invasive treatment with few complications and has a wide variety of clinical applications, and it is one of the main therapies for TN.^[4–6]^ Response rates of 77.5% to 97.6% are reported after RFT,^[4,7,8]^ with short-term efficacy of up to 100%,^[9]^ and the operation time under C-arm or computed tomography (CT) guidance is 15 to 55 min.^[10–12]^ Recently, RFT has been modified using the stereotaxic apparatus to reduce operation-related subsidiary injury.^[13]^ Nonetheless, in one study, 100% of patients developed facial numbness after RFT,^[14]^ and Tang et al^[6]^ have reported that 96% of patients with TN of mandibular division (V3) experienced masticatory atonia. Cases of blindness have also been reported.^[6,15]^

Previous animal experiments have shown that temperatures of ≤80°C damaged Aδ and C unmyelinated nerve fibers, but had no impact on Aα and Aβ nerve fibers.^[16–18]^ However, upon clinical application of RFT at 75°C to 80°C, a large number of patients experienced facial numbness, masticatory atonia, and other complications, which is not concurrent with the preclinical findings.^[5]^ There has been controversy in the recent literature regarding the suitable temperature for RFT in TN.^[19,20]^ Temperatures ranging from 60°C to 95°C have been recommended, but excessive temperatures will unavoidably cause serious complications. Lack of standardization can easily lead to misunderstanding and confusion among clinicians.^[19]^ Furthermore, because of the specificity of ophthalmic division of trigeminal nerve (V1), the recommended temperature and treatment protocols for RFT in TN for V1 are different from those of RFT for TN of maxillary division (V2), V3, and V2+V3, but several studies have failed to investigate TN of V1 separately.^[8,9,21]^ Also, idiopathic trigeminal neuralgia (ITN) and secondary trigeminal neuralgia (STN) have not been strictly distinguished in some previous studies, and the long-term pain relief was better for ITN than for STN.^[21,22]^ Therefore, RFT for TN needs further in-depth study.

The aim of this study was to compare pain relief, complications, and patient satisfaction during long-term follow-up in patients with bilateral TN after bilateral RFT at different temperatures (75°C and 68°C) on each side.

## 2 Materials and methods

### 2.1 Patient population

This was a randomized prospective cohort study. Patients with bilateral ITN of V2, V3, and V2+V3 who underwent RFT in the Department of Pain Management at Shengjing Hospital of China Medical University and Daqing Oilfield General Hospital from May 2011 to April 2016 were enrolled. Patients who underwent treatment at V1 and those with TN of V1 were excluded. Treatment was randomized according to the random number table. The treatment temperature was chosen firstly for the left side and then the different temperature was used for the right side. For example, if 68°C was selected for the left side according to the random number table, the right side was treated at 75°C, and vice versa. Idiopathic trigeminal neuralgia was diagnosed according to the international headache society (IHS)-II (2008) and IHS-III (2013) diagnostic criteria,^[23,24]^ and inclusion criteria for the study were as follows: age >28 years; preoperative pain score >5 on visual analogue scale (VAS); poor therapeutic results or serious adverse reactions after trials of conventional medical treatments before RFT; agreed to RFT and signed written informed consent form; and aware of the possible complications of RFT including facial numbness and masticatory atonia.

Exclusion criteria were as follows: diagnosis of STN (including TN secondary to herpes zoster, multiple sclerosis, intracranial tumors, or intracranial space-occupying lesions, but not excluding vascular compression at the root of trigeminal nerve); poor physical status, including severe heart, lung, liver, and kidney disease, severe hypertension, diabetes, or coagulation disorders; and compromised mental status or unwilling/unable to undergo RFT due to the risk of complications.

Patients were offered RFT after at least 3 months of conventional medical treatment with carbamazepine, gabapentin or pregabalin, and magnetic resonance imaging (MRI) of the trigeminal nerve and the head was routinely performed.

The study was approved by the Ethics Committee of Shengjing Hospital of China Medical University.

### 2.2 Radiofrequency thermocoagulation technique

Radiofrequency thermocoagulation was performed under CT guidance. The patients were lying supine on the CT bed, with vital signs monitored routinely. Puncture points were marked at 2.5 to 3 cm lateral to the angles of the mouth bilaterally, and the puncture pathways were then plotted. Local anesthesia with 0.5% lidocaine was then administered and a 22-G 10-cm puncture cannula with a 5-mm active tip was advanced along the puncture pathway to the foramen ovale, with the puncture direction monitored by CT and adjusted as needed to ensure that the needle entered the foramen ovale. When needle placement was confirmed by CT along with reactive pain in the innervation zone of the trigeminal nerve, and no blood or cerebrospinal fluid (CSF) return was observed, the RF electrode was connected for testing. After a discharge-like pain in the innervation zone of the trigeminal nerve was induced at 50 Hz 1 millisecond 0.1 to 0.2 V, 1.5% lidocaine 0.2 mL was injected for local anesthesia and the temperature was gradually increased from 50°C to the preset value within 240 seconds, followed by RFT (68°C or 75°C) for 180 seconds. If the patients continued to feel local pain, the position of the needle tip was adjusted and the RFT was repeated. The puncture needle was withdrawn after RFT, and the contralateral RFT at the different temperature was performed according to the same procedure. Patients were sent back to the wards postoperatively.

### 2.3 Follow-up

The follow-up investigators were blinded to both the surgery and the patients. All patients received follow-up by telephone, outpatient visit, or family visit once per month within the first 6 months and thereafter once every 3 months. Postoperative pain relief, complications, and patient satisfaction with RFT by side were recorded. Preoperative pain was scored by VAS, and postoperative pain relief was evaluated with the Barrow Neurological Institute (BNI) pain scale^[25]^ as follows: I (excellent), complete pain relief requiring no drugs; II, mild pain not requiring drugs; III, moderate pain requiring drugs for complete control; IV, moderate pain requiring drugs and not completely controlled; and V, the pain was severe or not relieved. Barrow Neurological Institute scores of I to III indicated satisfactory pain relief, whereas BNI IV and V indicated poor pain relief, which also served as a criterion to evaluate recurrence after RFT. The initial BNI score was obtained at discharge.

Facial numbness was evaluated using the BNI facial numbness scale, as follows: I, no numbness; II, mild numbness; III, moderate numbness; IV, severe numbness.

Patient satisfaction was evaluated with a five-step scale^[26]^ as follows: I, poor; II, fair; III, neutral; IV, good; and V, best. Scores of I, II, and III meant the patients found the treatment unsatisfactory while scores of IV and V indicated satisfactory results.

### 2.4 Statistical analysis

Under normal distribution and equality of variances, a pair-wise *t* test was used to analyze the quantitative data; otherwise a 2-sample Wilcoxon Rank-sum test was used. The ranked data were analyzed by Wilcoxon Mann–Whitney test, and the rate data by χ^2^ test. The follow-up data were analyzed using Kaplan–Meier curves and log-rank test, and the effects of relevant factors using Cox regression. Censored follow-up was defined as patient death, loss of follow-up, or secondary invasive intervention after recurrence, and the censored point was the last visit. All data were analyzed using IBM-SPSS 19.0 software (Armonk, NY: IBM Corp). *P* < 0.05 suggested that a difference was statistically significant.

## 3 Results

### 3.1 Patient characteristics

The patient characteristics are shown in Table 1. A total of 62 patients with ITN met the inclusion criteria. There was no difference in the affected nerve divisions between 2 sides (*P* > 0.05), the pre-RFT pain duration was 10.5 ± 6.2 months, and the pre-RFT pain score (VAS) was 7.5 ± 1.8. All patients regularly used carbamazepine, gabapentin, or pregabalin for pain management. Fifty-six patients completed the follow-up. Two patients were lost to follow-up and 4 died. These 6 cases were all regarded as censored cases. The hospital duration and follow-up duration of patients were 5.24 ± 1.3 (4–8) days and 32.4 ± 15.6 (8–60) months.

**Table 1:**
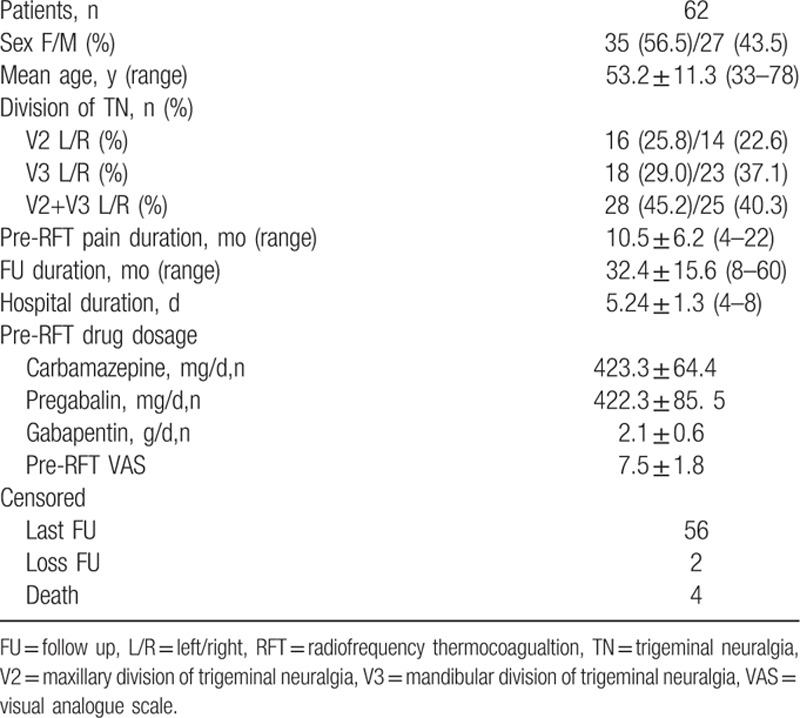
Patient characteristics.

### 3.2 Intraoperative and in-hospital data and complications

The intraoperative and in-hospital data are shown in Table 2. All patients successfully completed RFT, and operative times were 26.3 ± 5.4 minutes and 28.4 ± 4.9 minutes, respectively. Mouth penetration occurred in a total of 8 cases during local anesthesia or puncture. In all of these, RFT was continued after replacement of the puncture needle. There were 24 cases (11 and 13 in 2 groups, respectively) of unilateral facial swelling. Five patients had bilateral facial swelling; all were treated with cool compress and recovered within 1 week. There was no difference between sides in the incidence of blood or CSF return after puncture of the foramen ovale. Seven patients had postoperative nausea and vomiting and 3 had headache and dizziness with intracranial hypotension, which were relieved after symptomatic treatment and bed rest. Facial numbness, corneal hypoesthesia, and masticatory atonia occurred at both temperatures, and partial cases recovered at 2 to 3 days. Facial numbness persisted at discharge on the side undergoing 68°C RFT in 8 (12.9%) patients and at the side undergoing 75°C RFT in 49 (79.0%) patients, and corneal hypoesthesia and masticatory atonia also occurred more frequently at 75°C (*P* < 0.05). Some patients had no relief on either side at postoperative day 3, possibly due to failure of the surgical procedure, and all of these underwent secondary RFT with complete relief and no difference between sides. No patient had decreased hearing, deafness, or blindness, and there were no lethal complications. All patients were satisfied with RFT at the time of discharge.

**Table 2:**
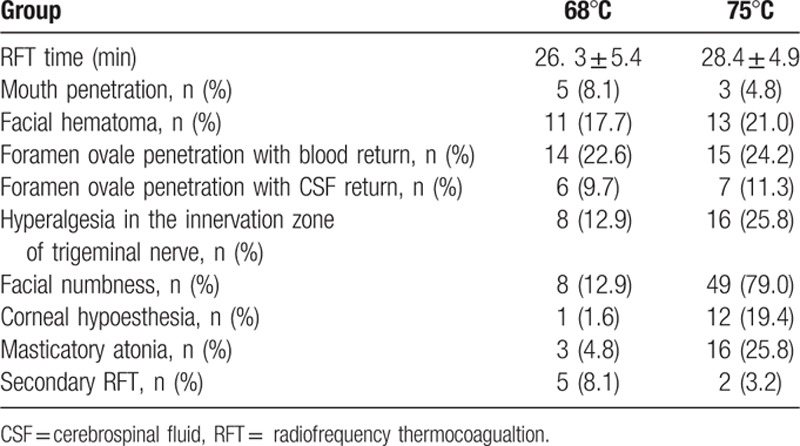
Intraoperative and in-hospital duration data.

### 3.3 Long-term follow-up data

#### 3.3.1 Pain relief

**Figure 1:**
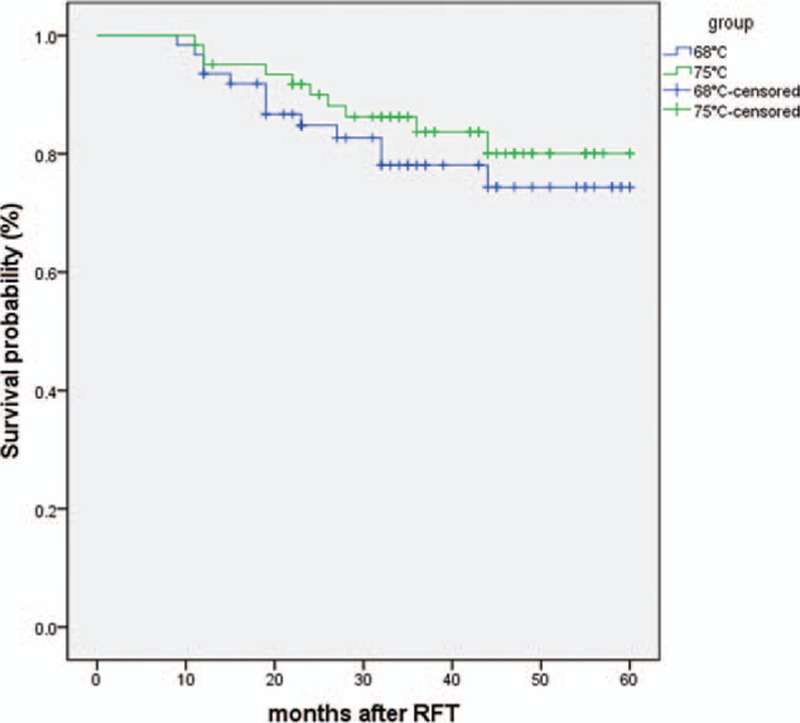
Kaplan–Meier curve shows the patients without any pain (Barrow Neurological Institute score I) after radiofrequency thermocoagulation (RFT). Group 1 = 68°C group, group 2 = 75°C group.

Among 62 patients, 56 completed the 5-year follow-up. Four patients died and 2 were lost to follow-up. The censored point for these 6 patients was the last visit. Pain relief was satisfactory at discharge in all cases. The BNI score was I (excellent) at 75°C in 99.2% of patients and in 98.2% at 68°C at discharge, 95.1% and 93.5% at 1 year, 84.3% and 78.1% at 3 years, and 80.7% and 74.4% at 5 years. The finding of greater pain relief at 75°C was not statistically significant (Fig. 1). During follow-up, 13 and 10 patients experienced recurrence and 6 patients had bilateral recurrence. The frequency of recurrence was 17 case-times in total. Seven patients underwent secondary RFT, nerve block, or other surgical interventions based on their own wishes, and the censored point for these patients was the last visit. There was no statistically significant difference in outcomes between treatment temperatures in these patients.

#### 3.3.2 Postoperative complications

During long-term follow-up (Table 3), 49 (79.0%) patients had postoperative facial numbness after RFT at 75°C, of which 51.6% of cases were scored as mild (BNI score II), 24.2% as moderate (BNI score III), and 3.2% (2 patients) as severe. The patients with severe numbness also had severe pain, and their recovery time was 12.7 ± 7.9 (8–22) months. There were 8 patients with facial numbness at 68°C, including 7 (11.3%) patients with mild numbness (BNI score II) and 1 (1.6%) with moderate numbness (BNI score III). All of these patients recovered within 1 year (6.4 ± 3.9 [4–12] months), and none had severe numbness. The differences in the incidence and severity of facial numbness between the 2 temperatures were statistically significant (*P* < 0.05). Finally, there were 6 patients with bilateral facial numbness.

**Table 3:**
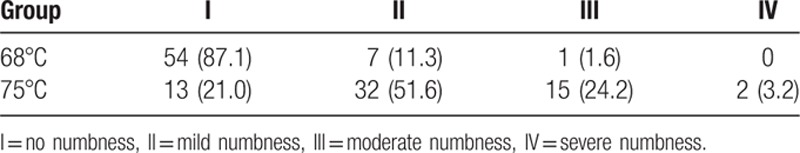
Facial numbness complications.

Masticatory atonia occurred in 16 patients at 75°C and in 3 at 68°C; recovery times were 14.3 ± 9.4 (7–28) months and 6.6 ± 4.6 months, respectively. Three patients with masticatory atonia had not recovered by the end of follow-up and 3 had bilateral masticatory atonia. The differences in the incidence and recovery time of masticatory atonia between the 2 temperatures were significant (*P* < 0.05).

Twelve patients had corneal hypoesthesia after RFT at 75°C; all were recovered after 11.8 ± 6.2 (6–18) months. In addition, 1 patient had bilateral corneal hypoesthesia (75°C and 68°C), which recovered within 4 months. The difference in recovery time between 2 sides was significant (*P* < 0.05).

No blindness, deafness, intracranial hemorrhage, other serious complications, or RFT-related deaths occurred in any patient during the 5-year follow-up.

#### 3.3.3 Patient satisfaction

**Figure 2:**
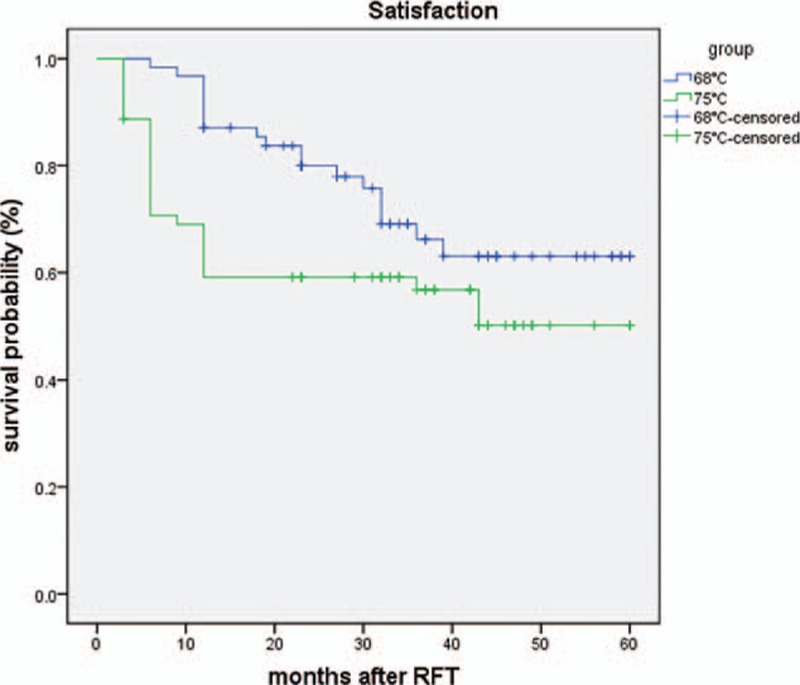
Kaplan–Meier curve shows the satisfaction of idiopathic trigeminal neuralgia (ITN) patients after radiofrequency thermocoagulation (RFT). Group 1 = 68°C group, group 2 = 75°C group.

Figure 2 shows patient satisfaction with RFT by side. At discharge, all patients were satisfied with bilateral RFT. In the long-term follow-up, the level of patient satisfaction decreased as more patients had late complications and recurrence, so that at 1 year, treatment was scored as satisfactory in 69.0% and 90.3% (75°C and 68°C) of patients, in 56.8% and 66.2% at 3 years, and in 50.1% and 63.6% at 5 years. The difference was statistically significant (*P* < 0.05; Log Rank 0.44, Breslow 0.006, Tarone–Ware 0.016).

## 4 Discussion

Different temperatures have been selected for RFT treatment of TN in various reports,^[14,19,20]^ which may create confusion and misunderstanding among clinicians, and may cause clinicians to assume that only higher temperatures will yield better pain relief, in some cases inducing them to perform 10-minute RFT at temperatures of up to 95°C, with unavoidable occurrence of serious complications. In most studies, RFT is performed at 70°C to 95°C, and it is agreed that RFT is safe and effective for treatment of TN. However, these studies have focused mainly on overall treatment results of RFT, whereas the effects of different temperatures have not been investigated. This study was designed to compare efficacy, safety, and patient satisfaction after RFT at 75°C (a clinically common temperature)^[5,6,27]^ and 68°C (a lower temperature) in cases of bilateral ITN, with the final purpose of providing definite guidance for clinicians. In order to exclude the effects of individual subjectivity on pain evaluation, the study was performed in patients with bilateral ITN and RFT was performed bilaterally at different temperatures on each side. Our study results showed that there was consistent pain relief at both 68°C and 75°C, while there were fewer complications and greater patient satisfaction at 68°C compared with 75°C.

All patients had pain relief after RFT at both temperatures, and 99.2% and 98.2% of patients rated pain relief as excellent at 75°C and 68°C, respectively, immediately after RFT. At the end of the 5-year follow-up, the percentage of patients absolutely without pain was 80.7% and 74.4%, respectively, and there was no statistically significant difference. The efficacy of RFT at both temperatures is coincident to that in previous studies^[28–30]^ and even better than that reported in several other studies.^[21,31,32]^ Improved efficacy for pain relief in the current study might be associated with the following factors: as opposed to previous reports^[4,14,22,33]^ that have investigated ITN and STN together, we strictly included ITN and excluded STN; we included V2, V3, and V2+V3 for RFT comparison but excluded V1 and V1 TN because RFT at a lower temperature, nerve block, or pulse RFT (PRFT) is required for V1, and thus V1 and V1 TN should be addressed separately. However, V1, V2, and V3 have been investigated together in many studies; RFT parameters were also influential. In this study, the stimulation voltage was set as 0.1–0.2 V and the procedure was performed under CT guidance. It is known that RFT can produce a better effect at lower stimulation voltages and in a position closer to the nerve,^[34]^ and RFT under CT guidance is more accurate than RFT under C-arm guidance.^[35]^ There were no puncture failures in our study, while a failure rate of 4% has been reported for C-arm-guided RFT procedures.^[36]^

During follow-up, 10 and 13 patients had recurrence, and 6 patients had bilateral recurrence; the difference in recurrence rates (16.1% at 75°C and 21.0% at 68°C) was not statistically significant, and this is consistent with the results of a previous trial of RFT at 75°C to 85°C for treatment of TN at V2 and V3.^[6]^

We found that there is no significant difference in the efficacy of RFT at 68°C and 75°C RFT, and there was no statistically significant difference in the recurrence rate between the 2 temperatures. However, the rate of complications associated with RFT at 75°C was significantly higher. The rate of facial numbness after RFT at 75°C was 79.0%. Among these cases, 15 (24.2%) patients had moderate facial numbness (BNI score III) and 2 (3.2%) had severe facial numbness (BNI score IV) that affected their quality of life. The severity of facial numbness at 75°C in our study is less than that reported by Tang et al,^[6]^ who adopted RFT at 75°C to 85°C, and the incidence of facial numbness at 75°C is also lower than that in previous reports, including one report of RFT at a higher temperature in which 98% of patients had facial numbness^[37]^ and a report by Lai et al^[5]^ in which 87.8% of patients had facial numbness after RFT at 75°C for treatment of recurrent TN. There were 8 (12.9%) patients who had mild facial numbness after RFT at 68°C in the current study, and this rate is also lower than that previously reported for RFT at a higher temperature.^[6,27]^

Approximately 25.8% of patients had masticatory atonia after RFT at 75°C, as compared with a rate of masticatory atonia of 96% in a previous study of RFT at 75°C to 85°C for V3^[6]^ as well as with other studies reporting higher rates of masticatory atonia at higher temperatures.^[16,38]^ This finding indicates that RFT at temperatures >75°C may increase the risk of masticatory atonia. Meanwhile, the rate of masticatory atonia was only 4.8% at 68°C, which was far lower than that after RFT at a higher temperature and is consistent with that reported by Wael,^[22]^ who performed RFT at 60°C to 70°C.

The rate of corneal hypoesthesia of 19.4% after RFT at 75°C in the current study is consistent with previous results.^[22,39]^ However, the rate of corneal hypoesthesia was much lower (1.6%) after RFT at 68°C. In our study, the differences in the incidences of facial numbness, masticatory atonia, and corneal hypoesthesia according to treatment temperature were all statistically significant, but there were no serious complications (e.g., blindness, deafness, and intracranial hemorrhage) at either temperature.

Patient satisfaction is widely assessed during the postoperative evaluation and can reflect patient acknowledgment of surgical efficacy.^[40–42]^ In this study, all patients were satisfied with RFT at both temperatures at the time of discharge, perhaps because moderate or severe pain was immediately relieved after RFT. However, as more and more patients experienced complications and recurrence during the long-term follow-up, there was a decrease in patient satisfaction. Patient satisfaction after RFT at 75°C was 69.0% at 1 year and 50.1% at 5 years, and this was significantly lower than that after RFT at 68°C. Diminishing patient satisfaction after RFT at 75°C was thought to be mainly because of complications and symptom recurrence. The complications of facial numbness, masticatory atonia, and corneal hypoesthesia are associated with postoperative discomfort or reduced quality of life, and thus would be expected to cause decreased satisfaction in affected patients after RFT. This finding suggests that postoperative complications and patient satisfaction should be taken into account during the pursuit of greater pain relief.

The major perioperative complications of RFT were facial hematoma, nausea and vomiting, and headache with dizziness and other symptoms of intracranial hypotension syndrome. Although all of these complications were successfully treated, we believe they had an impact on the evaluation of immediate post-RFT efficacy. We instructed patients to gradually reduce oral drugs (e.g., carbamazepine) that they had been using preoperatively during several days postoperatively before discontinuing them. Therefore, the long-term follow-up was started at discharge, while in previous reports^[14,16,21,22]^ the follow-up was started immediately after operation, which influenced the accuracy of postoperative observation. There were 5 and 3 patients who had no pain relief or rated outcomes as unsatisfactory during the postoperative hospital stay, and all of them had secondary RFT before discharge. We noted that all of these patients were elderly, and we speculated that the poor results may have been because they were unable to provide timely feedback regarding pain relief in the trigeminal innervation zone due to slow response times or verbal communication disorders. All 8 patients seemed to have complete relief after the secondary RFT procedure, and therefore we felt that it was reasonable to include them in the long-term follow-up.

Patients with bilateral ITN were included in the study so that we could compare results of bilateral RFT at different temperatures. However, this may have contributed to some bias in patient satisfaction, that is, in cases where persistent unilateral facial numbness may have caused dissatisfaction with the entire procedure. Additionally, bilateral RFT did not permit evaluation of patient quality of life by side (by unilateral RFT), which is a shortcoming of this study.

## 5 Conclusion

In summary, we comparatively observed pain relief, complications, and patient satisfaction after treating patients with bilateral ITN by bilateral RFT at different temperatures. Our study results showed that there was consistent pain relief after RFT at both 68°C and 75°C, but RFT at 68°C was superior to RFT at 75°C in terms of the rate and severity of complications and in terms of patient satisfaction. Therefore, clinicians should consider performing RFT at lower temperatures so as to reduce complications, and a temperature of 68°C can be recommended.
